# Analysis and design of randomised clinical trials involving competing risks endpoints

**DOI:** 10.1186/1745-6215-12-127

**Published:** 2011-05-19

**Authors:** Bee-Choo Tai, Joseph Wee, David Machin

**Affiliations:** 1 Department of Epidemiology and Public Health, Yong Loo Lin School of Medicine, National University of Singapore, Singapore; 2 Department of Radiation Oncology, National Cancer Centre, Singapore; 3 Children's Cancer and Leukaemia Group, University of Leicester, UK

## Abstract

**Background:**

In randomised clinical trials involving time-to-event outcomes, the failures concerned may be events of an entirely different nature and as such define a classical competing risks framework. In designing and analysing clinical trials involving such endpoints, it is important to account for the competing events, and evaluate how each contributes to the overall failure. An appropriate choice of statistical model is important for adequate determination of sample size.

**Methods:**

We describe how competing events may be summarised in such trials using cumulative incidence functions and Gray's test. The statistical modelling of competing events using proportional cause-specific and subdistribution hazard functions, and the corresponding procedures for sample size estimation are outlined. These are illustrated using data from a randomised clinical trial (SQNP01) of patients with advanced (non-metastatic) nasopharyngeal cancer.

**Results:**

In this trial, treatment has no effect on the competing event of loco-regional recurrence. Thus the effects of treatment on the hazard of distant metastasis were similar via both the cause-specific (unadjusted *csHR *= 0.43, 95% CI 0.25 - 0.72) and subdistribution (unadjusted *subHR *0.43; 95% CI 0.25 - 0.76) hazard analyses, in favour of concurrent chemo-radiotherapy followed by adjuvant chemotherapy. Adjusting for nodal status and tumour size did not alter the results. The results of the logrank test (*p *= 0.002) comparing the cause-specific hazards and the Gray's test (*p *= 0.003) comparing the cumulative incidences also led to the same conclusion. However, the subdistribution hazard analysis requires many more subjects than the cause-specific hazard analysis to detect the same magnitude of effect.

**Conclusions:**

The cause-specific hazard analysis is appropriate for analysing competing risks outcomes when treatment has no effect on the cause-specific hazard of the competing event. It requires fewer subjects than the subdistribution hazard analysis for a similar effect size. However, if the main and competing events are influenced in opposing directions by an intervention, a subdistribution hazard analysis may be warranted.

## Background

In a randomised, double-blind, three-period clinical trial of lisinopril in patients with chronic heart failure [1], factors associated with different modes of cardiovascular death were investigated to guide physicians in their treatment decisions. In this trial, sudden death was considered as a competing risk for chronic heart-failure death, and hence it was important to distinguish between factors that were associated with increased mortality and factors which were simply markers of a worse prognosis.

Similarly, in trials designed to delay or avoid irradiation among children with malignant brain tumour, although irradiation following disease progression is an important event, competing events include declining radiotherapy (RT) following disease progression or elective RT despite no evidence of disease progression. In order to accurately describe the cumulative need for RT and evaluate how each event contributes to the delay or advancement of irradiation in such instances, it is vital to account for these competing events via a competing risks analysis [2,3].

In such trials, it is commonplace to summarise the competing risks outcomes using the Kaplan-Meier (KM) method of survival analysis. However, the KM method does not evaluate how each event contributes to the overall failure. Besides, it relies on the stringent assumption of independence between different event types and overestimates the event-specific failure probabilities.

In this paper, we describe how clinical trials involving competing risks outcomes may be analysed and designed using data from a randomised clinical trial (SQNP01) of patients with nasopharyngeal cancer (NPC) as illustration [4].

## Competing Risks in Cancer Studies

Under the classical competing risks framework, a subject may be simultaneously exposed to several distinct events, but may eventually only fail from one of these. In such settings, the occurrence of a specific event would preclude the competing risks from being observed. In cancer clinical trials for example, the main outcome is usually death (*D*), although local recurrence (*R*), distant metastasis (*M*) and second malignancy (*S*) are always of relevance. For some patients the full path from randomisation to death can be recorded. However, if *D *occurs first, then only the time to death from randomisation, *t*_D, _will be recorded, and the times to the other events *t*_R_, *t*_M _and *t*_S _will not be observed. Similarly, if *M *occurs before the other events are observed, then this may potentially initiate a change in therapeutic strategy and hence change the course of the disease. Thus, in cancer clinical trials, the first event is usually of interest, and as such, competing risks modelling focus on the occurrence of the first event even in cases where multiple events (for example, local recurrence followed by distance metastasis) can occur. This is because the additional complexity of analysing such data does not often yield a materially different conclusion [5,6].

## Illustrative Trial

The objective of the SQNP01 trial was to evaluate the role of chemo-RT and adjuvant chemotherapy using combination chemotherapy comprising cisplatin (CDDP) and 5-Fluorouracil (FU) with RT in treating patients with locally advanced NPC [4]. All patients received a standard course of RT to a dose of 70Gy in 35 fractions. For patients randomised to receive chemo-RT followed by adjuvant chemotherapy (CRT), three cycles of concurrent CDDP (25 mg/m^2^/d for 4 days) were administered on weeks 1, 4 and 7 of RT. A further three cycles of adjuvant chemotherapy comprising CDDP (20 mg/m^2^/d for 4 days) and 5-FU (1,000 mg/m^2^/d for 4 days) were administered between weeks 11 and 19.

The primary outcome of this trial was overall survival, and the trial was designed on the basis of detecting a difference in absolute survival at 2 years of 25%. This assumed a survival rate was 55% for RT alone and 80% for CRT, a two-sided test size of 5% and a power of 90%. In this randomised clinical trial, distant metastasis was considered to be an important secondary outcome because it has been shown that about 50% of patients with very large and/or supraclavicular lymph nodes will relapse distantly [7] even after a full course of irradiation.

**Figure 1:**
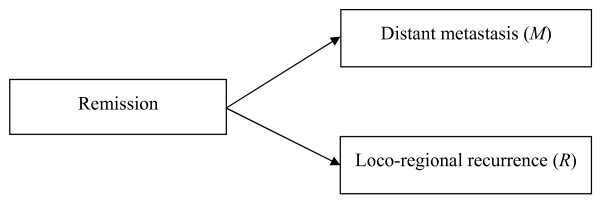
**A competing risks model for the SQNP01 trial**.

For simplicity of illustration, we consider distant metastasis (*M*) as the main event of interest, and only one competing risk, loco-regional recurrence (*R*). The latter includes relapses at the primary site and the neck. If we consider only the first event that occurs, such a classification fits naturally into the competing risks framework, where a subject may only fail from one of these two causes (Figure 1).

## Methods

### Statistical Terminology

#### Kaplan-Meier event-free survival (EFS) estimates

In cancer clinical trials, a patient may experience treatment failure as a consequence of the recurrence of the primary disease at the local site or development of distant metastases. The event free survival (*EFS*) time, *t*_*j*_, is often measured from the date of randomisation to the date of first occurrence of any of these failure types.

Assuming these events to be independent, the event-free survival probability may be estimated as

The probability of being event-free at time *t*_*j*_, *EFS*(*t*_*j*_) is calculated from *EFS*(*t*_*j*__-1_), the probability of being event-free at *t*_*j*__-1_, *n*_*j *_the number of patients who are free from any event just before *t*_*j *_and *d*_*j *_the number of events at *t*_*j*_.

#### Cause-specific hazard function

The type of failures is sometimes described using the cause-specific hazard function, *h*_*l*_(*t*_*j*_), which in the context of competing risks, can be separately estimated for each event type *l*, as follows

Here *n*_*j *_is the number of patients who are event-free just before *t*_*j *_and *d*_*lj *_the number who fail from event *l *at *t*_*j*_.

#### Cumulative incidence or subdistribution

The Cumulative Incidence (*CMI*), also referred to as a subdistribution, estimates the event-specific probability of each event, in the presence of all other competing risks [8]. For an event of type *l*, it is estimated by

and may be expressed as

For large time, the subdistribution's limit is the prevalence of the event of interest.

#### Logrank and Gray's tests

The logrank test is widely used for comparing cause-specific hazards between groups of clinical interest. It censors the competing events at the time of occurrence of the main event. Considering two treatments for example RT and CRT in the SQNP01 trial, this test assumes the null hypothesis *h*_*l, RT *_(*t*) = *h*_*l, CRT *_(*t*), and hence a hazard ratio (*HR*) of 1. The *HR *may be estimated by

where *O*_*CRT *_and *E*_*CRT *_refers to the observed and expected number of events amongst patients receiving CRT and *O*_*RT *_and *E*_*RT *_are the corresponding events for those receiving RT. Since the estimate of *HR *is not normally distributed, its 95% confidence interval (CI) may be estimated via log *HR *assuming [9].

The Gray's test [10] has been advocated to compare the cumulative subdistribution hazard that has a one-to-one relation with the cumulative incidence. The subdistribution hazard function is defined by

#### Incorporating prognostic factors

In the SQNP01 trial, nodal status and tumour size could also influence survival outcomes, and hence it is important to account for them when evaluating the extent of treatment difference. Various regression models have been proposed to relate the cause-specific hazard or the *CMI *to covariates [11-13]. We discuss two proportional (namely the cause-specific and subdistribution) hazard models for analysing competing risks data.

#### Cause-specific Cox model

Suppose for simplicity, we consider two event types, a main event of interest, Event *M*, and a competing risk, Event *R*, with a set of *p *covariates, *x*_1_, ..., *x*_*p*__. _The Cox proportional hazards model which is frequently used for modelling the hazard of a single failure may be extended to model the cause-specific hazard for Event *M *as follows:(1)

where *h*_0__*M*_(*t*) is the baseline hazard of Event *M*, and exp(*β*_*M*__1_), exp(*β*_*M*__2_), ..., exp(*β*_*Mp*_) are the cause-specific hazard ratios (*csHR*) which measure the effect of the respective covariates on Event *M*, taking the competing event, Event *R*, as censored [8]. The cause-specific hazard for Event *R *is similarly formulated.

#### Modelling subdistribution hazards

It has been argued that the cause-specific Cox analysis is not adequate for modelling competing risks data because it censors the competing events [14]. Such censoring is assumed to be non-informative, and this procedure fails to consider that those who have experienced a competing event can never experience the main event of interest. As the effect of covariates on the cause-specific hazards does not translate to an effect on the cumulative incidence, Fine and Gray have proposed modelling of the *CMI *based on the proportional subdistribution hazards model [11]. This model allows the incorporation of the effects of multiple risk factors, whereas the Gray's test [10] considers the impact of only a single prognostic factor. Based on the subdistribution hazard model, if an individual experiences the competing event instead of the main event, it is assumed that he remains in the risk set forever, and so his main event time is infinite.

The subdistribution hazard model is formulated in a similar manner as the cause-specific Cox model shown earlier, except that the exponential of the regression coefficients now denote the subdistribution hazard ratios (*subHR*) of the respective covariates on the subdistribution hazard of event *M*, for example.

#### Sample size estimation

Sample size estimation procedures for both the cause-specific and subdistribution hazard models are based on the Schoenfeld formula for the Cox model [15]. Two key parameters need to be specified: (i) planned hazard ratio (*HR*_Plan_) that quantifies the treatment effect, and (ii) anticipated proportion of failures from the main event of interest (Ψ_Plan_).

Assuming an experimental (CRT) and a standard (RT) treatment, the benefit of CRT over RT for the event of interest may be expressed in terms of either *subHR *or *csHR*, depending on the choice of statistical model. The total number of events, *e*, assuming Type I and Type II errors of *α *and *β *respectively, can be expressed as(2)

where *z*_*γ *_denotes the upper *γ*-quantile of the standard Normal distribution, *p*_*CRT *_the proportion randomised to receive CRT and ln *HR*_Plan _the natural logarithmic form of the anticipated effect size. In the absence of censoring, the total sample size, *n*, equals the number of (say type *M*) events observed, while in the presence of censoring,(3)

### Estimation based on subdistribution hazard model

The Schoenfeld formula [15] has been extended to estimate sample size for modelling *CMI *via the subdistribution hazard by Latouche *et al*. [16] If there were no censored observations, Ψ_Plan _reduces to the *CMI *at time *t *for the main event, that is *CMI*_*M*_(*t*), in the SQNP01 trial. In the presence of censoring, Ψ_Plan _may be estimated by (1 - *c*)*CMI*_*M*_(*t*), where *c *is the anticipated proportion of censored observations [16].

### Estimation based on cause-specific hazard model

Pintilie proposed the Cox model to estimate sample size when testing the effect of a covariate on the cause-specific hazard in the presence of competing risks [17]. The times to the main and competing events were assumed to be independent and exponentially distributed. In the context of a randomised clinical trial, it is further assumed that treatment affects the cause-specific hazard of the main but not the competing event [17,18].

The anticipated probability of the main event *M*, Ψ_Plan_, may be expressed in terms of the hazard functions by(4)

where *λ*_*M *_denotes the cause-specific hazard of main event *M*, *λ *the total hazard of both events, *a *the accrual duration and *f *the additional follow-up period.

As Pintilie has shown [17], the cause-specific hazard for our main event *M *may be derived from the *CMI *as follows:(5)

where *CMI*_*M *_and *CMI*_*R *_represent the *CMI *of the respective events at time *T*, the total study duration. The cause-specific hazard for the competing event, *λ*_*R *_can be similarly obtained.

The sample size may be estimated as before, but the treatment effect is now expressed by ln *csHR *when estimating *e*, the expected number of events. Pintilie [17] and Machin *et al*. [19] have developed software for estimating sample size and power based on the cause-specific hazard method.

Schulgen et al. [20] have proposed a similar method to estimate the sample size for clinical trials involving time-to-event competing risks outcomes based on a multi-state model, assuming a time-homogeneous Markov process. The probability of observing an event of type *M *by time *t *is given by

This is essentially equation (2) of Pintilie [17], the cumulative incidence function of *M *in the presence of competing risks. Based on the cumulative incidence probability of the event of interest and the cumulative incidence probability of the competing risks, Pintilie [17] obtained the solution of the system for the cause-specific hazard as shown in equation (5) above. As the formulation of the anticipated probability in Schulgen et al [20] is the same as that presented by Pintilie and is as shown in equation (4) above, and with both methods assuming constant cause-specific hazard rates, we expect the two approaches to yield the same sample size estimates when the same parameters are specified in the estimation.

## Results

**Figure 2:**
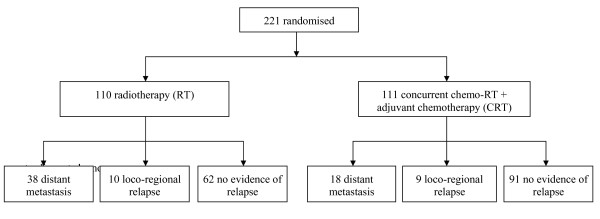
**Progress of 221 patients enrolled in the SQNP01 trial**.

Between September 1997 and May 2003, a total of 221 patients were randomised to receive RT alone (*n *= 110) or CRT (*n *= 111) [4]. Relapse at first site were documented in a total of 75 patients: 19 loco-regional and 56 distant metastases (Figure 2). In particular, distant metastasis (event *M*) occurred in 38 patients who received RT and 18 CRT. Ten patients in RT as compared with 9 in CRT experienced loco-regional recurrence (event *R*).

### Estimating cause-specific hazard and cumulative incidence

In Table 1, we illustrate computational details of the cause-specific hazard as well as the cumulative incidence for event *R *amongst those receiving CRT of the SQNP01 clinical trial. The time to event *R *is denoted in column (2). For example, the first subject experienced this event at Day 153 post treatment, while for the second subject, it occurred at Day 248. Tied occurrences of event *R *were not reported, and so *d*_*Rj *_= 1 at each event time. We further estimate the cause-specific hazard, *h*_*R*_(*t*_*j*_), by dividing *d*_*Rj*_, the number in column (3) by *n*_*j*_, the number in column (4). In column (7), we obtain *CMI*_*R*_(*t*_*j*_) = *CMI*_*R*_(*t*_*j*__-1_) + *EFS*(*t*_*j*__-1_) × *h*_*R*_(*t*_*j*_). For *j *= 9, *CMI*_*R*_(*t*_9_) = *CMI*_*R*_(*t*_8_) + *EFS*(*t*_8_) × *h*_*R*_(*t*_9_) = 0.0760 + 0.7131 × 0.0667 = 0.1235.

**Table 1:** Cumulative incidence for event *R *(loco-regional recurrence) of the SQNP01 trial

*j* (1)	*t*_*j *_ (2)	*d*_*Rj *_ (3)	*n*_*j *_ (4)	*h*_*R*_(*t*_*j*_) (5)	*EFS*(*t*_*j*__-1_) (6)	*CMI*_*R*_(*t*_*j*_) (7)
0	0	0	111	0.0000	1.0000	0.0000
1	153	1	109	0.0092	1.0000	0.0092
2	248	1	100	0.0100	0.9261	0.0184
3	274	1	95	0.0105	0.8888	0.0278
4	277	1	94	0.0106	0.8794	0.0372
5	295	1	93	0.0108	0.8701	0.0465
6	367	1	91	0.0110	0.8513	0.0559
7	399	1	86	0.0116	0.8326	0.0655
8	524	1	78	0.0128	0.8130	0.0760
9	1660	1	15	0.0667	0.7131	0.1235

### Comparing logrank and Gray's tests

Using the logrank test to evaluate the treatment effect on the cause-specific hazards, a beneficial effect of CRT on event *M *was suggested (*HR *= 0.43, 95% CI 0.25 - 0.72; *p *= 0.002). However, the treatment effect was not detected for event *R *(*HR *= 0.84, 95% CI 0.34 - 2.07; *p *= 0.711) (Table 2).

**Table 2:** Cause-specific hazard ratio (HR) comparing CRT versus RT for each competing event based on the logrank test

Variables	*O*	*E*	Unadjusted HR (95% CI)	*p*-value
***M*: Distant metastasis**				
RT	38	26.54	0.43 (0.25 - 0.72)	0.002
CRT	18	29.46	1.00	
				
***R*: Loco-regional recurrence**				
RT	10	9.19	0.84 (0.34 - 2.07)	0.711
CRT	9	9.81	1.00	

**Figure 3:**
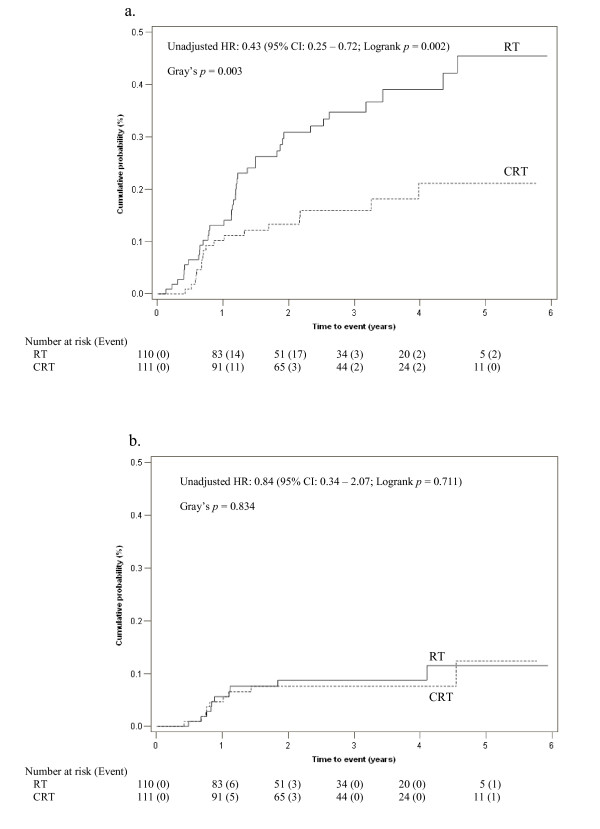
**Cumulative incidence of relapse amongst patients in RT (solid line) and CRT (dashed line) for (a) Event *M*: Distant metastasis, (b) Event *R*: Loco-regional recurrence**. HR = Hazard Ratio; CI = Confidence Interval.

The 2-year cumulative incidence of event *M *was notably lower in the CRT arm as compared with RT (13.3% versus 30.8%). Consistent with the logrank test, the Gray's test showed a beneficial treatment effect on event *M *in favour of CRT (*p *= 0.003) (Figure 3). However, the 2-year cumulative incidences of event *R *were similar for both treatments (8.8% versus 7.6%). Again, the result of Gray's test (*p *= 0.834) concurred with the logrank test.

### Cause-specific hazard analysis

The impact of other patient related factors cannot be readily accounted for using the logrank test. Hence, since known risk factors influencing survival outcomes of the SQNP01 trial include nodal status and tumour size, we further adjust for these prognostic variables via the cause-specific Cox model. Table 3 shows that the unadjusted *csHR *estimates comparing treatment for both the logrank test and cause-specific Cox model do not differ appreciably. The unadjusted *csHR *and adjusted *csHR *estimates suggest reduced hazards of distant metastasis among patients randomised to receive CRT (Tables 3 and 4). Adjusting for nodal status and tumour size did not alter the estimates materially (unadjusted *csHR *= 0.43 versus adjusted *csHR *= 0.39).

**Table 3:** Logrank and Cox cause-specific hazard ratios (*csHR*), subdistribution hazard ratios (*subHR*) and associated 95% confidence intervals for evaluating the treatment effect

	Unadjusted *csHR*		Unadjusted *subHR*
	
Competing event	Logrank	Cox	Subdistribution
*M*: Distant metastasis	0.43 (0.25 - 0.72)	0.43 (0.24 - 0.75)	0.43 (0.25 - 0.76)
			
*R*: Loco-regional recurrence	0.84 (0.34 - 2.07)	0.84 (0.34 - 2.08)	0.91 (0.37 - 2.24)

**Table 4:** Cox cause-specific hazard ratios (*csHR*), subdistribution hazard ratios (*subHR*), and associated 95% confidence intervals for evaluating the treatment effect; adjusted for nodal status and both tumour size.

	Adjusted for nodal status and tumour size	
	
Competing event	*csHR*	*subHR*
*M*: Distant metastasis	0.39 (0.22 - 0.71)	0.42 (0.23 - 0.79)
		
*R*: Loco-regional recurrence	0.69 (0.27 - 1.79)	0.81 (0.30 - 2.17)

As for loco-regional recurrence, there was no evidence of treatment effect (unadjusted *csHR *= 0.84; 95% CI 0.34 - 2.08). Although an attenuated effect was observed after adjusting for nodal status and tumour size (adjusted *csHR *= 0.69; 95% 0.27 - 1.79), statistical significance was not achieved for this comparison. Thus, in terms of the rate of tumour progression, patients who received CRT have lower rates of distant metastasis and loco-regional recurrence.

### Subdistribution hazard analysis

In the SQNP01 trial, it is important to quantify the proportion of NPC patients who experienced distant metastasis in order to target therapeutic strategy at this common cause of failure. As such, the subdistribution hazard which directly quantifies the proportion rather than the cause-specific hazard which describes the rate in which a patient develop distant metastasis, would be more appropriate for addressing this objective. The subdistribution hazards analysis was implemented in STATA version 11 using the command **stcrreg **[21]. As before, we adjust for nodal status and tumour size when assessing treatment effect on the cause-specific subdistribution hazards. The unadjusted results considering only treatment are also presented for comparison in Table 3.

In keeping with the cause-specific hazard analysis, the subdistribution hazard analysis also suggested significant reduction in subdistribution hazard for event *M *amongst those receiving CRT (unadjusted *subHR *0.43; 95% CI 0.25 - 0.76), and no treatment effect on event *R *(unadjusted *subHR *0.91; 95% CI 0.37 - 2.24) (Table 3). This is consistent with our analysis comparing *CMI *via the Gray's test, which showed reduction in *CMI *of *M *(13.3% versus 30.8%) amongst patients receiving CRT and no difference in *CMI *of *R *(7.6% versus 8.8%) between the two groups (Figure 3).

For event *M*, the results remain unaltered after adjusting for nodal status and tumour size, with adjusted *subHR *= 0.42 (95% CI 0.23 - 0.79) (Table 4). In the case of event *R*, a larger but non-significant effect was observed (adjusted *subHR *= 0.81; 95% CI 0.30 - 2.17). This implies that patients on CRT were less likely to have distant metastasis, but the beneficial effect of CRT on loco-regional recurrence was not evident.

### Designing a randomised clinical trial with competing risk outcomes

#### Planning

For the expository purpose of illustrating the methods for designing a clinical trial with competing risks outcomes, we suppose at the study planning, the investigator postulated that the treatment effect on event *M *was similar in magnitude to what was found in the literature, based on the SQNP01 trial. He further assumes equal allocation between treatments (ie. *p *= 0.5), and plans for a power of 80% and a two-sided test of 5% corresponding to *Z*_0.2 _= 0.84 and *Z*_0.025 _= 1.96 from the standard Normal table.

#### Subdistribution approach

Deciding a priori that the subdistribution hazard analysis will be implemented, and anticipating that CRT would reduce the hazard of event *M *with *subHR *= 0.43 as in Table 3, then applying equation (2), the number of events required is

Suppose the investigator assumes the 5-year *CMI *of event *M *is similar to that observed in the SQNP01 trial, that is, *CMI*_*M *_= 0.35, *CMI*_*M+R *_= 0.47, and so the censored cases *c *= 1 - 0.47 = 0.53 at 5-year. From these, Ψ_Plan _can be estimated by (1 - *c*)*CMI*_*M *_= 0.47 × 0.35 = 0.1645. Thus based on equation (3), the total required sample size is

#### Cause-specific approach

Suppose the investigator chooses instead to measure the effect of treatment on the main event *M *using the cause-specific hazard, and assumes *csHR *= 0.43 which is similar in magnitude to *subHR *(Table 2). Using equation (2), the expected number of events remains unchanged, that is *e *= 45.

We further assume that the 5-year *CMI *of event *M *in RT and CRT are 0.45 and 0.2 respectively, and that of event *R *in both groups is 0.12, with accrual duration *a *= 4 years, additional follow-up period *f *= 1 year, and total study duration, *T *= *a *+ *f *= 5 years. To estimate, Ψ_Plan_, the probability of the main event *M *in the RT group, we derive its cause-specific hazard from the 5-year *CMI *using equation (5):

We derive *λ*_*M*, *CRT *_= 0.0482, *λ*_*R*, *RT *_= 0.0355 and *λ*_*R*, *CRT *_= 0.0289, similarly. Since *λ*_*RT *_= 0.1333 + 0.0355 = 0.1688, from equation (4), the expected proportion of failures due to the main event *M *in the *RT *arm is

Similarly, the expected proportion of failures due to the main event *M *in the *CRT *arm is

Thus, Ψ_Plan _= p_CRT _Ψ_Plan, CRT _+ (1- p_CRT_) Ψ_Plan, RT _= 0.5(0.3047) + 0.5(0.1271) = 0.2159.

From equation (3), the total sample size required via the cause-specific hazard analysis is thus

This is notably less than the size of the trial which was designed assuming a subdistribution hazard model.

#### Sample size estimation for other effect sizes

Table 5 illustrates sample size for a range of effect sizes corresponding to those observed for nodal status and tumour size in patients with locally advanced NPC. The number of events and samples sizes required increase rapidly for a range of effect sizes for *subHR *from 0.39 to 0.75. Although similar effects were observed for both analyses, a larger sample size was required for the subdistribution hazard analysis.

**Table 5:** Total number of events required, *e*, and total sample size, *n*, for detecting various effect sizes for Event *M *(distant metastasis)

Variables	Cause-specific hazard			Subdistribution hazard		
	
	*csHR*	*e*	*n*	*subHR*	*e*	*n*
						
Nodal status (Absent, Present)	0.40	38	153	0.39	38	231
Treatment (CRT, RT)	0.43	45	209	0.43	45	274
Tumour size (1, 2-4)	0.71	268	1209	0.75	379	2304

## Discussion

Under the classical competing risks framework, a subject may be simultaneously exposed to several distinct events, but may eventually only fail from one of these. In such settings, the occurrence of a specific failure type would preclude the competing events from being observed. The cumulative incidence estimates have been advocated to summarise competing risks data [5,8,9,12,22]. This approach appropriately accounts for each competing risk, and provides an unbiased estimate in terms of event-specific probability.

In randomised clinical trials involving competing risks, the treatment effect on the cause-specific hazard is sometimes evaluated via the logrank test. However, its use is controversial [23,24]. This method censors the competing events at the time of occurrence of the main event. It also assumes that treatment has no effect on the hazards of competing risks [17,18]. Thus its use may only be appropriate if the objective is to measure the effect of treatment on a specific failure, in isolation of other competing events.

In practice, the cumulative incidence curves are more relevant for explaining the relative impact of therapies to a patient. Using the SQNP01 trial data, the competing risks method found 13% of patients in CRT developing distant metastasis at 2-year as compared to 31% in RT. The 2-year cumulative incidence of loco-regional recurrence hovered around 8% for both treatments. In these instances, the results of both logrank and Gray's tests concurred.

The subdistribution hazard model estimates the effect of treatment on the *CMI *of each event, while incorporating the effects of other relevant covariates. In trials designed to evaluate the role of treatment on disease recurrence, it is important to evaluate the contribution to failure by each event. The *CMI *which quantifies the cause-specific probability provides a more direct measure of the overall failure rate than the cause-specific hazard.

Consistent with the assumption of no treatment effect on the hazards from competing risks when implementing the cause-specific hazards analysis [25], we found the two models to concur well for the SQNP01 data. A beneficial effect of CRT on the cause-specific hazard was observed for distant metastasis, but it had no effect on the competing event of loco-regional recurrence. In this instance, both the cause-specific and subdistribution hazard analyses showed CRT reduces the hazard of distant metastasis by the same extent (57%).

Nevertheless, it should be noted that differences between the cause-specific and subdistribution hazard ratios may be appreciable if the main and competing events are influenced in opposite directions by an intervention [3,26,27]. Williamson *et al*. [24] have shown that in such instances, the Gray's test comparing *CMI *has greater power than the log-rank test comparing the cause-specific hazard to detect treatment differences, and so a subdistribution hazard analysis may be warranted.

The choice between the two models has implications on the study design. As reported by Latouche and Porcher [18] and illustrated using the SQNP01 data, the subdistribution hazard analysis requires many more subjects than the cause-specific hazard analysis even for the same magnitude of effect. As noted by Latouche *et al*. [16], the cause-specific hazard ratio differed from the subdistribution hazard ratio due to differences in the estimation of the cumulative incidence. For the former, it is based on 1 - Kaplan Meier survival estimates, which have been shown to overestimate the cumulative incidence [28]. The bias increases with time, and may be large for uncensored data, or for data whereby a large proportion of subjects fail from extraneous causes prior to the occurrence of the event of interest [8]. As such, differences in sample estimates between the two approaches are to be expected.

Although we have implemented the competing risks methodology specifically in nasopharyngeal cancer, it is widely applicable to other clinical settings such as epilepsy, Parkinson's disease or migraine, where more than one primary event may be of interest when evaluating the withdrawal of drug due to lack of efficacy and tolerability [24].

## Conclusions

The cause-specific hazard analysis may be appropriate when treatment has no effect on the cause-specific hazard of the competing event. However, if the main and competing events are influenced in opposing directions by an intervention, a subdistribution hazard analysis may be warranted. The design of clinical trials involving competing risks endpoints requires careful planning and the choice of the statistical models used should be made a priori to take into account such considerations.

## Abbreviations

5-FU: 5-Fluorouracil; RT: radiotherapy; CDDP: cisplatin; CI: confidence interval; CMI: CuMulative Incidence; CRT: chemo-RT followed by adjuvant chemotherapy; csHR: cause-specific hazard ratio; EFS: event free survival; HR: hazard ratio; KM: Kaplan-Meier; NP: nasopharyngeal cancer; subHR: subdistribution hazard ratio.

## Competing interests

The authors declare that they have no competing interests.

## Authors' contributions

All authors contributed to the design of the SQNP01 trial which was used as an illustrative example for this study. TBC performed all statistical analyses and drafted the first version of the manuscript in discussion with DM. All authors reviewed the manuscript critically, read and approved the final version.
